# Circular Estimate Method (CEM) - a Simple Method to Estimate *Caenorhabditis elegans* Culture Densities in Liquid Medium

**DOI:** 10.1186/s12575-018-0089-2

**Published:** 2019-01-15

**Authors:** Marcelo Estrella Josende, Silvana Manske Nunes, Larissa Müller, Marlize Ferreira-Cravo, José Marìa Monserrat, Juliane Ventura-Lima

**Affiliations:** 1 Instituto de Ciências Biológicas (ICB), Universidade Federal do Rio Grande - FURG, Av. Itália km 8, Rio Grande, RS 96203-900 Brazil; 2 Programa de Pós-Graduação em Ciências Fisiológicas (PPGCF) - FURG, Rio Grande, Brazil

**Keywords:** *Caenorhabditis elegans*, Nematode, Counting, Estimating, Accuracy, Precision, Reliability, Reproducibility

## Abstract

**Background:**

Nematodes are used in many different fields of science, including environmental and biomedical research. Counting and/or estimating nematode numbers is required during research. Although being one of the most common procedures, this apparently simple task is a time-consuming process, prone to errors and concerns regarding procedure, reliability, and accuracy. When an estimate is necessary, there is a traditional manual counting procedure that in this study it will be called as “drop method” (DM). This popular method that extrapolates an animal count from a small drop of fluid shows a high coefficient of variation. To solve this problem, the present study used the free-living nematode *Caenorhabditis elegans* to develop a new estimation procedure that was based on a relationship between area and volume of a larger sample.

**Results:**

The new method showed a low coefficient of variation and a close relationship between estimated and real counts of the total number of nematodes in large *C. elegans* suspensions. Reactive oxygen concentration was measured as an example of method application and to allow comparison between methods.

**Conclusion:**

The proposed method is accurate, facile and reproducible, requiring simple, inexpensive materials that make it an excellent alternative to the DM manual counting procedure. Although the DM is faster, its estimates are not as accurate or as precise as those of the new proposed method.

**Electronic supplementary material:**

The online version of this article (10.1186/s12575-018-0089-2) contains supplementary material, which is available to authorized users.

## Background

Nematodes are used for in many fields of science, including environmental and biomedical research, and are considered one of the most reliable, easy and assessable tools [1–7]. They are a diverse group of parasitic and free-living species that inhabit different environments and often are of socio-economic importance [8, 9]. Since its first description and characterization [10], *Caenorhabditis elegans* (*C. elegans*) has become one of the most researched model organisms, especially, since Brenner stressed its importance [11]. In the last 4 decades, *C. elegans*, as a model for biological research, has been pivotal in many different fields, such as biomedical, pharmacological and environmental toxicology [12–15]. Among its desirable feature are: a low maintenance cost; a very short period of development (approximately 72 h from egg to adult, depending on the temperature); a short lifespan (2–3 weeks); large amounts of offspring; and a sequenced genome [16, 17]. At present, it is considered an excellent model for understanding toxicological and parasitological studies [14, 18].

Nematode counting should be a simple task and ideally, be cheap, quick, accurate and reliable [19]. However, in practice, counting nematodes is a time-consuming process, prone to errors, which becomes a real challenge when researchers are faced with multiple samples [20, 21]. In the 1960’s, methods were suggested to standardize the manually counting of nematodes using counting slides/dishes [22, 23]. In the next decades, lots of different types of counting chambers were developed [24]. Although used, these were still considered too laborious [21]. Therefore, in the 1970’s, other methods were proposed to automate the counts [25–27]. Currently, new methods are still being suggested, aimed at simplifying this time-consuming task [20, 21, 28, 29].

When nematodes such as *C. elegans* are used in biological research, at least 2 counting procedures are usually performed, one of them before, and the other at the end of the experiment. The first measurement is used to determine the nematode culture density for each experimental treatment. This takes place just after the acquisition of a synchronized population, before the experiment at the chosen developmental stage for the study. The second counting procedure is performed at the end of the experiment, when the nematode progeny must be assessed, to get information about reproductive parameters. However, the procedure of counting and estimating nematode numbers, before and after an experiment, is basically the same.

The “drop method” (DM), as the name suggests, counts the number of nematodes that are in a drop (usually 10 μL) of a synchronized suspension of nematodes [30]. Based on 3–10 replicates, the average count is extrapolated to the volume of the nematode suspensions in the experiment [30]. An optimization of this protocol was recently published identifying sources of variability, such as uniform dispersion of the culture, priming of pipette tips and the location of the sampling within the container [31]. DM is the most popular method of estimate used by researchers around the world. However, in the present study, we demonstrate that DM had a low accuracy and reproducibility, illustrated by the high coefficient of variance (CV) between different replicates of the same sample.

Due to the inaccuracies of extrapolation, counts done on larger volumes should inherently be more accurate than those done on small volumes. Circular Estimate Method (CEM) uses a container that allows the count to be estimated from a 1/10 th volume of the culture. In the present study, we show that CEM describes a relationship between area and volume that allow an estimate to be drawn from a larger volume than with DM, with concurrent improvements in accuracy.

Therefore, the main objective of the present study was to develop a new method (CEM) to estimate *C. elegans* and other nematode species density/progeny, with higher accuracy and lower variability than the traditional method. As an example of how DM and CEM estimates could influence a biochemical assay, we tested the effect of using both methods on the results obtained from a reactive oxygen species (ROS) measurement. The main advantages of CEM were accuracy, precision, reproducibility, low cost and reliability.

## Results

In several studies with nematodes, the researcher needs to perform an accurate counting either to distribute the animals between different treatments or to obtain data about progeny and a variety of other parameters. However, this procedure is considered inconvenient and labour-intensive [29]. As an alternative to facilitate this procedure and still guarantee high accuracy and precision compared to the DM, the CEM was developed. In this section, the results obtained with CEM (which normally uses 5% of the total NSS volume or even more) were tested and compared to the results obtained with DM (which normally uses less than 1% of the total NSS volume). In routine laboratory proceedings, both CEM and DM are used for the purpose of estimation and not to generate nematode RCs in suspension. Therefore, the main findings presented and discussed in the present study were estimates extrapolated to samples. Comparative estimates of ENS and NSS were also performed and were presented.

### Time to Perform each Method (CEM and DM)

The mean time to perform the DM was shorter than for CEM. The mean time to count nematodes in each drop of DM (with approximately 100 worms) was approximately 1′ 30″. To do this for 5 replicates, calculate the mean and extrapolate the mean to the total ENS volume, took approximately 8′. To perform the CEM, as well as count the animals inside the circles, it was necessary to calibrate the software first and draw the circles.

The software calibration did not take longer than 20–25″. To calibrate the first circle to a radius of 2.435 mm and duplicate it as 3 other copies took 40–50″. Finally, the mean time to count the worms inside each circle (approximately 3 hundred worms) was approximately 3′ per circle. Therefore, the total time spent with CEM was approximately 14′ (1′ 30″ to calibrate the software and draw 4 circles, 12′ to count the nematodes in the 4 circles and finally 30″ to extrapolate the mean to the total area/volume).

### Variability Comparison of DM and CEM Methods

**Fig. 1:**
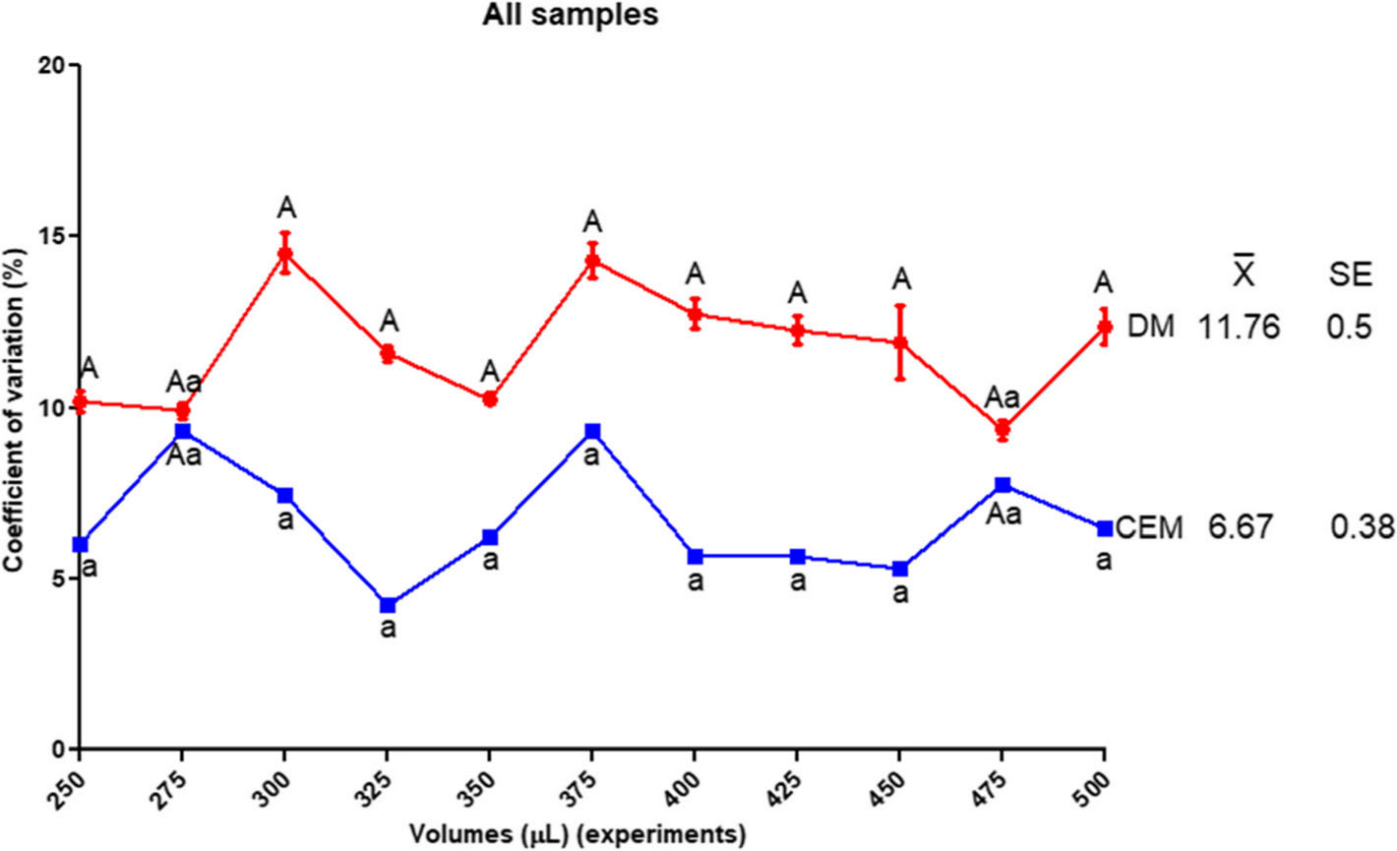
The coefficient of variation. Comparison among all the experiments (*n* = 12) showing a significant difference between methods (X ® = mean and SE = standard error). Different letters show significant statistical differences with respect to the sample volumes (α = 0.05)

**Fig. 2:**
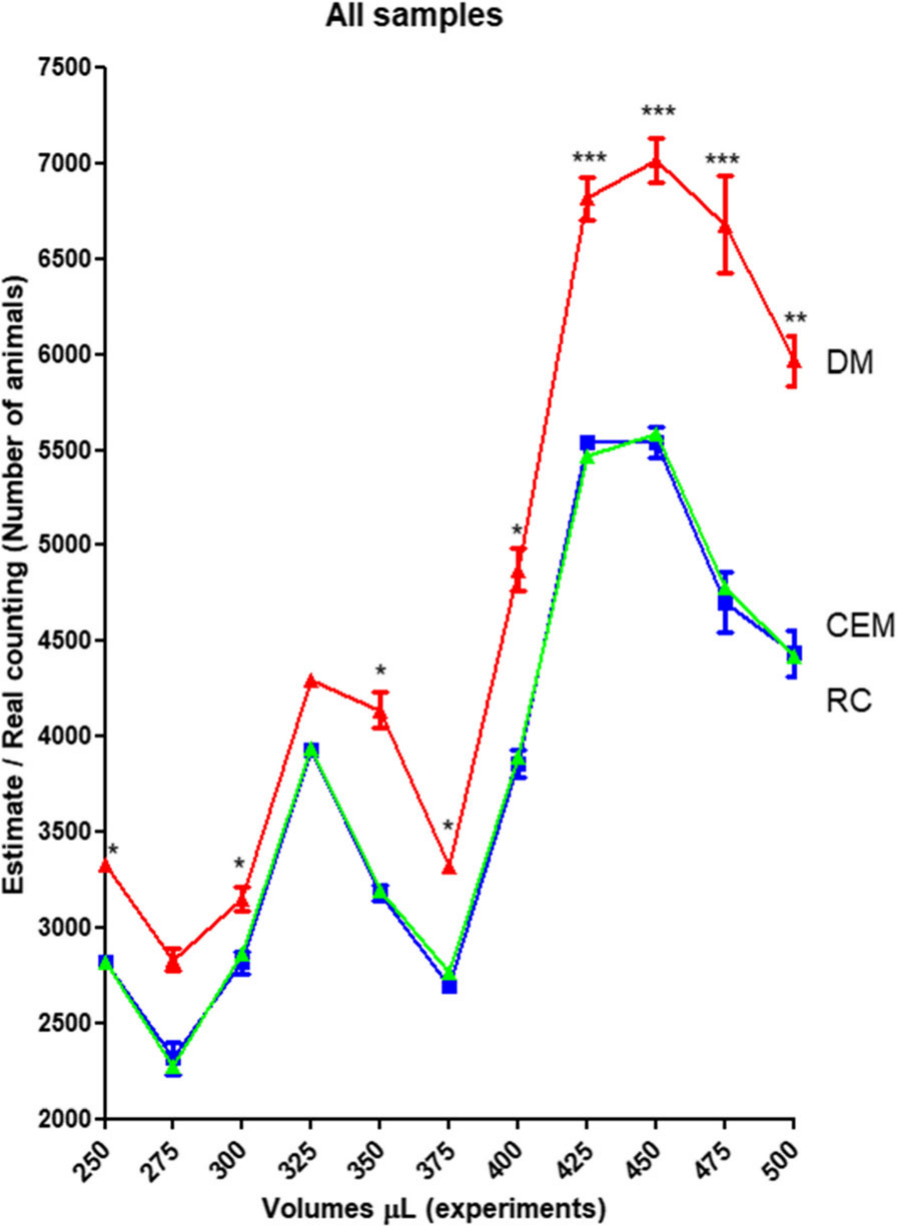
A comparison of all samples. Comparison of real counting (RC) of nematodes marked in green against estimates from DM (red) and CEM (blue; *n* = 12 for each volume assessed). The asterisks denote statistical difference between methods compared with the same volume experiment: more asterisks represent more significance in the difference (* = 0.01; ** = 0.001 and *** = 0.0001)

After 11 experiments performed in 2 replicates per experiment, the first statistical analysis was an overall comparison of CVs obtained from the samples. The CV for DM (9.35% ± 0.97 to 14.51% ± 2) was higher than for CEM (4.21% ± 0.46 to 9.31% ± 1.53), and its range of variation was greater too. The only 2 exceptions to this result were observed on the 275 μL and 475 μL samples (Fig. 1). DM showed a significant difference on most of the estimates performed in this study (Fig. 2 and Fig. 6).

### CEM and Comparison with RC

**Fig. 3:**
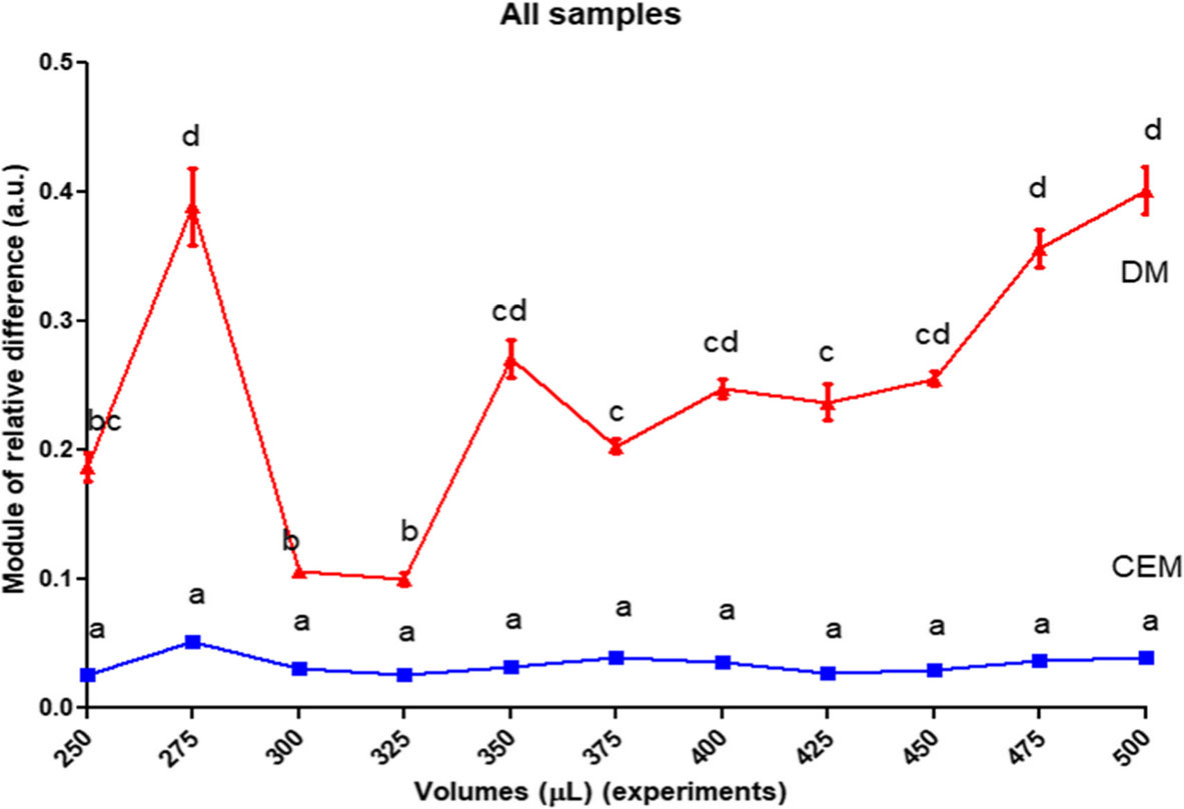
Modules of relative differences. Comparison between modules of relative differences (a.u., arbitrary units) obtained for each average estimation from CEM and DM, assuming for comparison purposes that the module of the RC is zero. Different letters show significant statistical differences (α = 0.05)

The CEM showed a better behaviour as an estimator, not only due to its smaller CV compared to DM (Fig. 1), but because it showed a closer relationship to RC values (Fig. 2). This finding became more evident after a comparison performed using the module of relative difference related to the RC (assumed as a value of zero, for comparison purpose; Fig. 3).

### Operators

**Fig. 4:**
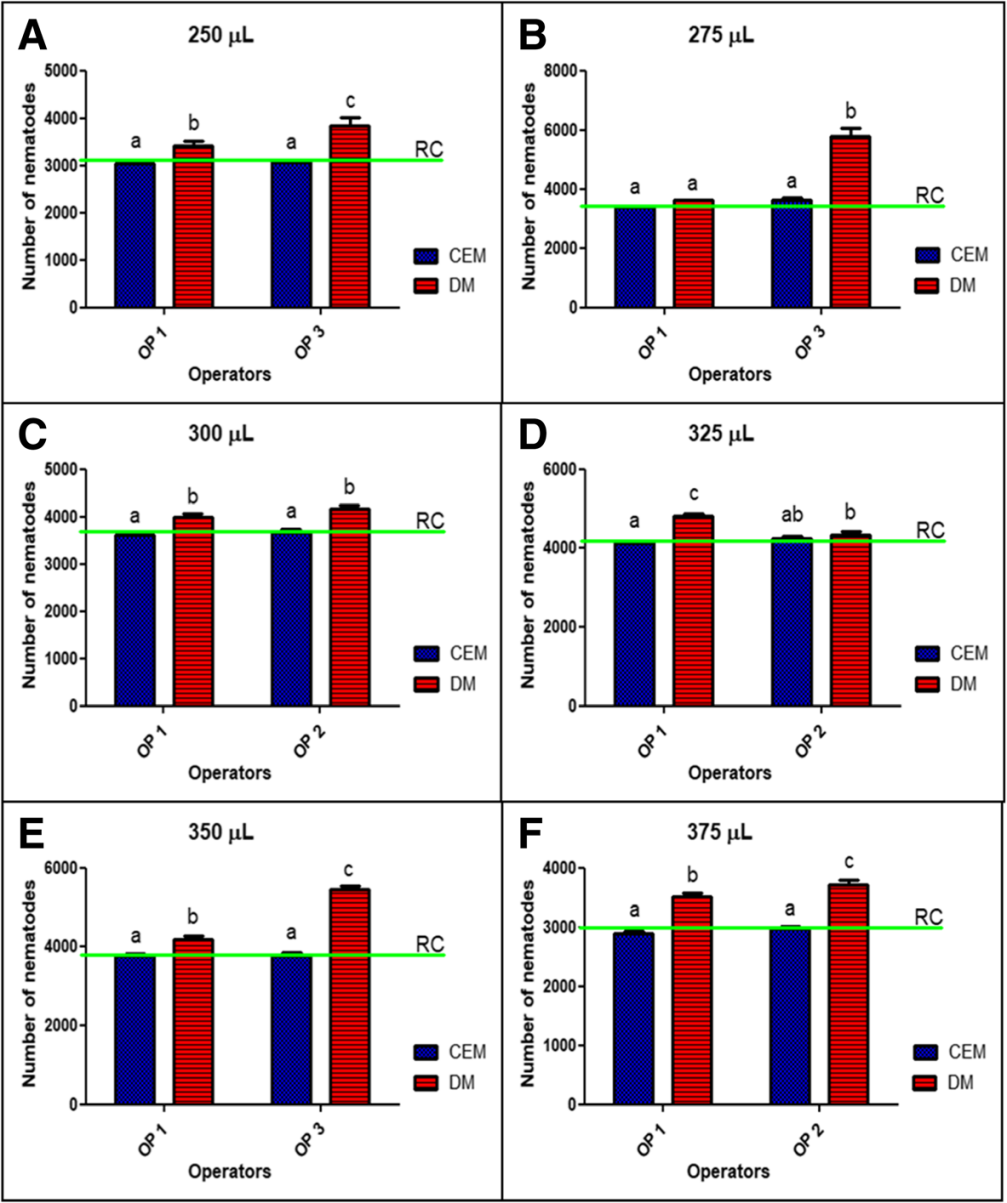
First set of sampled volumes (*n* = 6). Comparison between operators (OP 1, OP 2 and OP 3) and methods (CEM and DM) related to RC (green line) performed on 250 μL (**a**), 275 μL (**b**), 300 μL (**c**), 325 μL (**d**), 350 μL (**e**) and 375 μL (**f**). Different letters show significant statistical differences between operators compared to RC (α = 0.05)

**Fig. 5:**
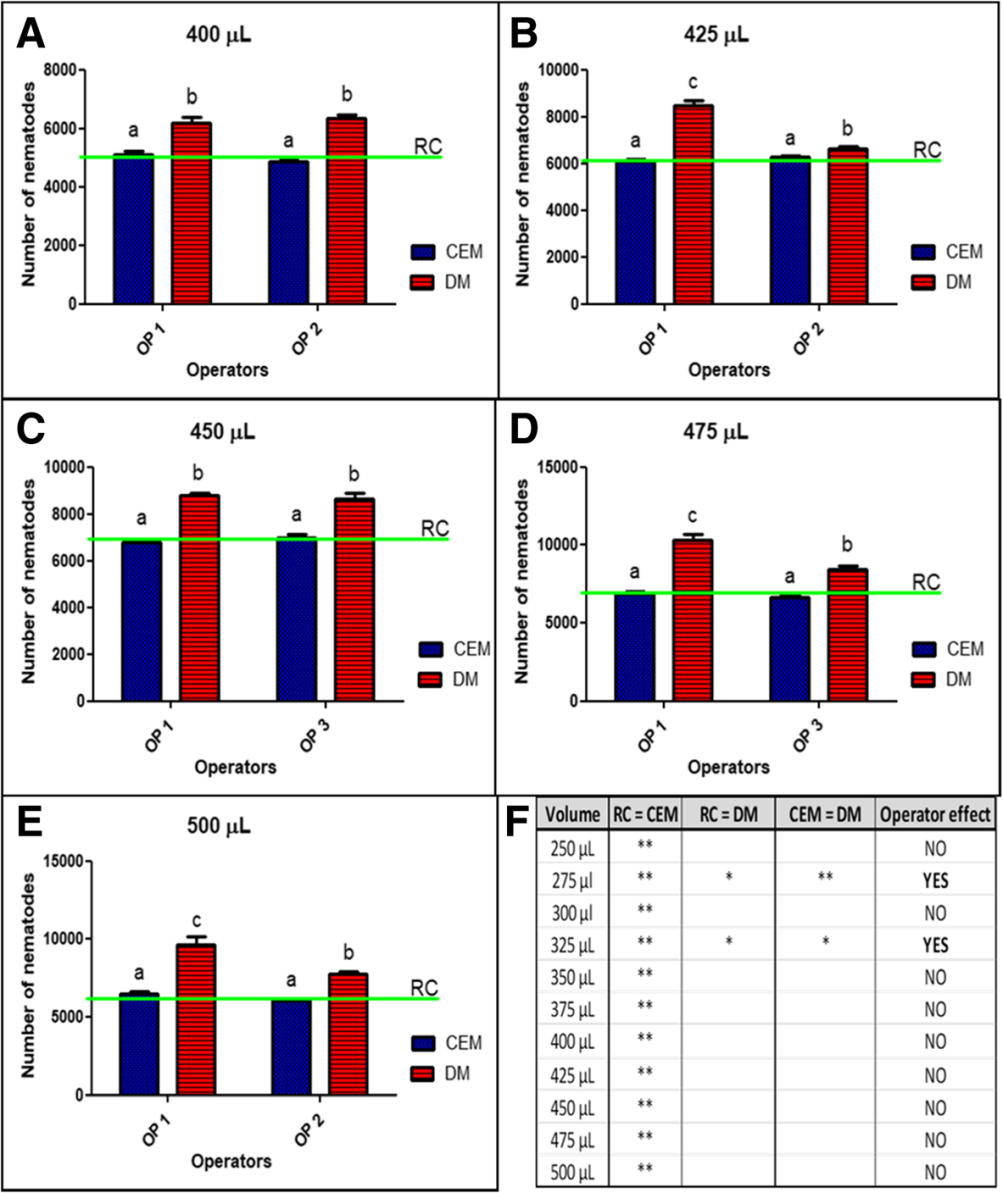
Second set of sampled volumes (*n* = 6). Comparison between operators (OP 1, OP 2 and OP 3) and methods (CEM and DM) related to RC (green line) performed on 400 μL (**a**), 425 μL (**b**), 450 μL (**c**), 475 μL (**d**) and 500 μL (**e**). Different letters show significant statistical differences between operators compared to RC (α = 0.05). Auxiliary Table (**f**) showing the operator effect of comparison between methods related to RC. Asterisks indicate to which condition one operator (*) or both (**) attended. First column: sample volume assessed. Second column: Conditional equality between RC and CEM. Third column: Conditional equality between RC and DM. Fourth column: Conditional equality between CEM and DM. Fifth column: Operator effect confirmation, assuming that DM was equal to RC or CEM (or both), at least for one operator, then the operator effect was present

The influence of operator (operator effect) was also assessed for both methods. With the exception of the 275 and 325 μL sample volumes (Fig. 4b and Fig. 4d), it was shown that operators did not introduce a statistical significant difference when CEM was compared to RC (Figs. 4 and 5). Figure 5 also shows an inner auxiliary table with the comparison among all sample volumes, confirming the effect the operators had on the close relationship between estimates performed with CEM and RC (Fig. 5f). The reference values used to plot the graphics in Fig. 4 and Fig. 5 are available in Additional file 1: Table SI.

### ROS

The ROS measurement is a very sensitive biochemical assay and a little difference in the sample adjustment can influence its results. The ROS measurement was performed as an example assay for comparison between DM and CEM. It was observed that the CEM and RC were statistical equally (978,978 A.U. ± 21,485, *n* = 6 and 958,785 A.U. ± 17,708, *n* = 3, respectively) while the DM (1,074,361 A.U. ± 23,838.68, n = 6) differed statistically both from CEM and the RC, thus stressing the influence of a non-accurate method (Fig. 7).

## Discussion

### Comparison of CEM and DM

The development of new techniques to improve the quality of assays is a challenge for all researchers. The majority of manual methods referenced here were performed using nematode counting dishes/slides, and as stated by Holladay et al., they came with the profound inconvenience of being time-consuming and error-prone [21].

For nematode estimation from a suspension, the DM is a good way when the researcher is using small volumes and relatively few animals in an experiment. However, if the researcher wants to estimate a large number of nematodes, such as in a population scan of soil nematode species on crops, an enzymatic assay or for RNA extraction [32–36], then CEM would be more reliable than DM. This is because CEM uses a larger sample size of 250–500 μL making the extrapolation less extreme than for DM, that uses 10 μL. By simple mathematics, an average 100 animals found in a 1/10 volume of a 10 ml NSS, for example, means there were 1000 animals in the 500 μL aliquot used for counting, or 20,000 nematodes in the total volume of NSS. Clearly, for DM counting the same NSS would involve a mean count of 20 animals per 10 μL. However, when the same NSS/ENS was estimated by CEM and DM, the total number of nematodes estimated after extrapolation was different for the 2 methods (Fig. 2 and Additional file 1: Table S1).

As mentioned above our explanation of this divergence was that 5% of the NSS was sampled with CEM, while only 0.1% is sampled with DM. The reason why the CEM count should be more consistent and closer to the values obtained with RC were self-evident (Additional file 1: Table S1).

The procedures that estimate the number of animals transferred among different systems, for example, from NSS to experimental containers or other plates, normally are not clearly stated [31]. Steps are not taken to validate estimates, even in routine experiments. This is partly due to the difficulty in performing such a validation, as it involves RCs of total volumes of NSSs. As an aid to such researcher, the present study demonstrated that the CEM was a more accurate and reliable estimator when compared with the DM in terms of the real counting for each sample, ENS or NSS (Fig. 2, Fig. 3a and b, respectively).

**Fig. 6:**
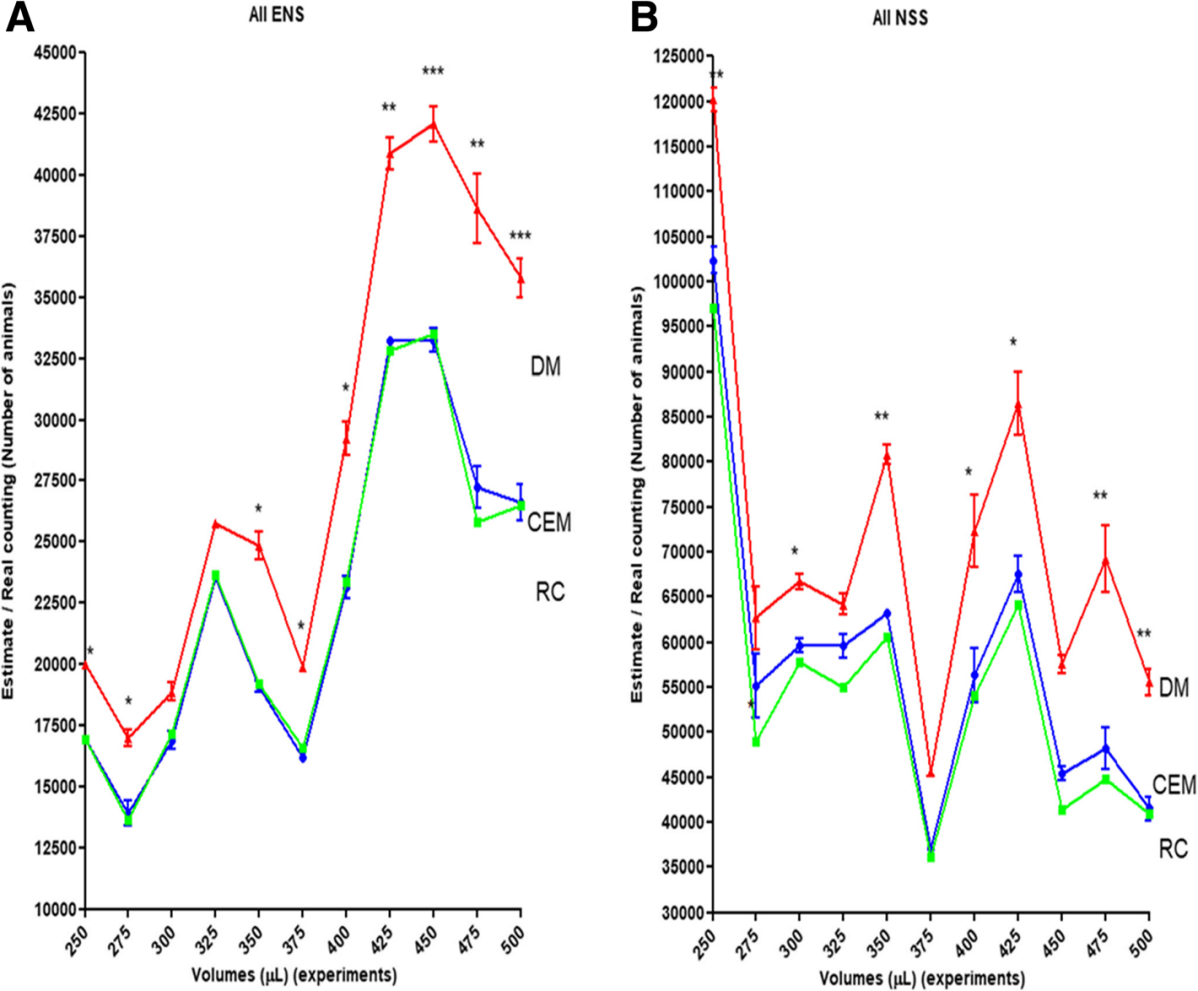
Estimates extrapolated to ENS (**a**) and NSS (**b**). Comparison among the real counting of nematodes (RC) marked in green against the estimates of DM (red) and CEM (blue; *n* = 12 for each volume assessed). The asterisks denotes statistical difference between methods compared with the same experimental volume, with more asterisks representing a more significant difference (* = 0.01; ** = 0.001 and *** = 0.0001)

The operator effect was also explored. All the volumes were assessed by operator 1 and compared to the volumes assessed by operator 2 and operator 3 (Table 1). An “Operator effect” was only found with volumes of 275 and 325 μL (Fig 4b and d). This means that in almost all cases, CEM was a better estimator than DM. Furthermore, the very close relation between CEM and RC observed in Fig. 2 was also found in Fig. 6, that brings information about the extrapolation of estimates from both methods to ENS (Fig. 6a) and NSS (Fig. 6b). In this sense, the observed “Operator effect” was not a determinant in favour to DM because in all cases the estimates performed with CEM were closer to the RC than DM.

**Table 1:** Parameters of the experiments

Operator	NSS volume	Sample volume	ENS volume
OP 1	10 mL	250 μL	1.5 mL
	8.01 ml	275 μl	1.65 mL
	5.96 ml	300 μl	1.8 mL
	3.76 mL	325 μL	1.95 mL
	8.02 mL	350 μL	2.1 mL
	5.52 mL	375 μL	2.25 mL
	2.87 mL	400 μL	2.4 mL
	6.67 mL	425 μL	2.55 mL
	3.72 mL	450 μL	2.7 mL
	5.77 mL	475 μL	2.85 mL
	4.32 mL	500 μL	3 mL
OP 2	6.97 mL	300 μL	1.8 mL
	6.03 mL	325 μL	1.95 mL
	4.77 mL	375 μL	2.25 mL
	7.77 mL	400 μL	2.4 mL
	3.55 mL	425 μL	2.55 mL
	4.87 mL	500 μL	3 mL
OP 3	8.05 mL	250 μL	1.5 mL
	2.93 mL	275 μL	1.65 mL
	6.15 mL	350 μL	2.1 mL
	3.65 mL	450 μL	2.7 mL
	3.5 mL	475 μL	2.85 mL

First column: the operator (OP 1, OP 2 and OP 3) responsible for each experiment. Second column: The total stock volume used as a base for the experiment. Third column: The aliquot used as a sample for CEM. Fourth column: The total experimental nematode suspension (ENS) assessed in each experiment

**Fig. 7:**
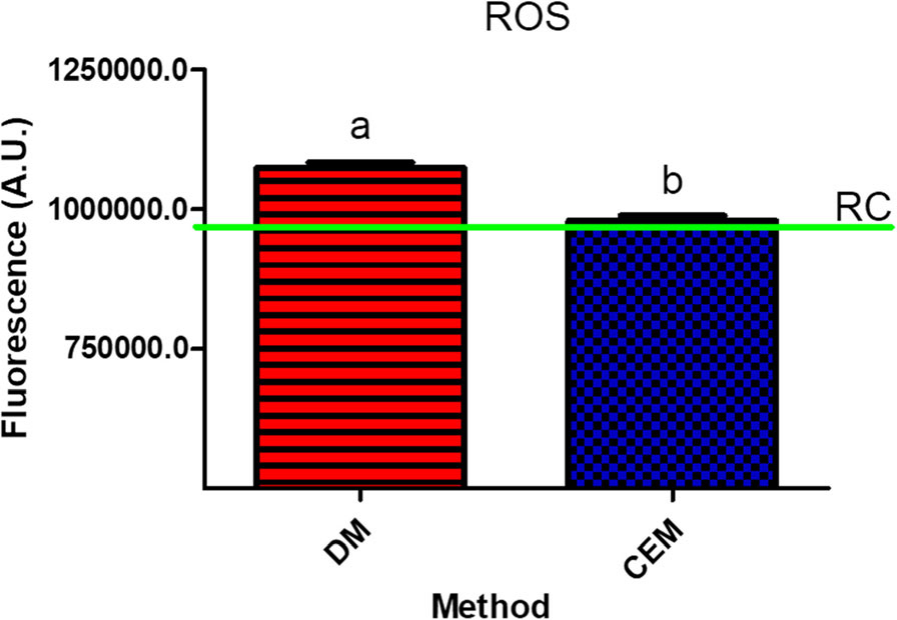
ROS measurements (*n* = 6). Comparison between methods (CEM and DM) related to RC (green line). The different letters denote statistical difference between methods

Finally, the ROS assay was chosen as an example to test the practical application of CEM and DM. According to our findings, the levels of ROS observed in the samples estimated by DM were statistical different from those obtained by CEM (Fig. 7). As previously mentioned, CEM and RC had a close relation (Fig. 2 and Fig. 3) and the results of RC and CEM did not demonstrate a statistical difference (Fig. 7). This example measurement of ROS served as a warning to those who want to perform biochemical assays with nematodes and use DM to estimate animal numbers.

### A Brief Discussion Comparing CEM and Other Automated Methods

According to Li et al., the procedure of manual counting nematodes is considered inconvenient and labour-intensive [29]. As an attempt to accelerate this task, other researchers have proposed high-throughput automated counting procedures [20, 21, 27, 29, 37].

**Fig. 8:**
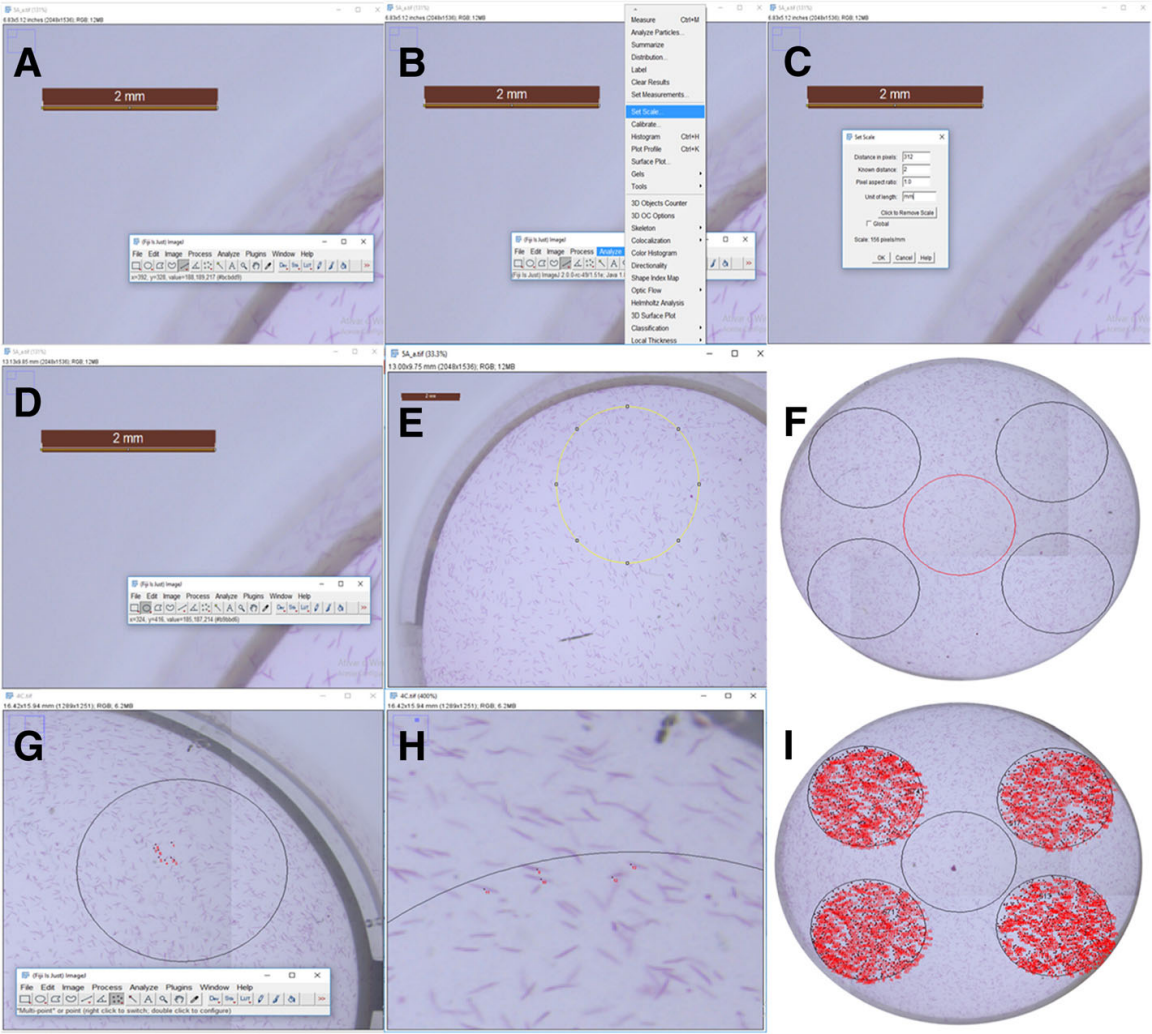
The sequence of steps in using ImageJ software. **a** Calibration of software. **b** Set of tools available for calibration. **c** The relation between pixels/mm and confirming of set scale. **d** Selection of oval/circular form. **e** Randomly placing the 1/10 counting circles. **f** Displacement of counting circles (black circles) and local reserved for homogenization (red circle at the centre). **g** Counting animals. **h** Selecting animals to be counted from those that are not (crossed animals). **i** Recording animal numbers for extrapolation to total NSS or ENS

One of the first automated counting procedures was a spectrophotometric method, with readings at 600 nm, used to study growth rates and increases in population size in suspensions of 30–40% sucrose solution [27]. Although this technique is simple and inexpensive, it is necessary to establish a standard curve for each new NSS by direct counting, which takes too much time [20]. The CEM method also needs to be calibrated, but the mathematics for this calibration is performed once, independently of the NSS density. By using the same brand of cellular culture plate with a consistent total area of well bottom surface, the 1/10 area represented as circles need only be calculated once. Another disadvantage of the method proposed by Patel and McFadden is that the light-scattering capacity of animals varies at different developing stages [27]. This is not a problem for CEM because the researcher can pick and count only animals at their chosen developmental stage (Fig. 8h).

Another procedure is based on the measurement of light transmittance through aqueous suspensions, measuring the absorbance at 550 nm with an ELISA microplate reader [20]. As for the previous method, a series of standard counts needs to be performed to construct a standard curve. Comparing the findings between this method and CEM, the main difference is that CM has a lower CV (4.21% ± 0.46–9.31% ± 1.53; Fig. 1) than this automated method (12–23%) [20]. Another factor that deserves attention is that the maximum number of worms counted using the CEM method is at least 3 times higher (> 6000 animals) than the method proposed by Robinson et al. (2000 animals) [20]. This means that even sampling a greater number of animals, CEM is a more accurate and precise method.

Another procedure using ImageJ software is the “High throughput nematode counting with automated image processing” method [21] that estimates nematode densities. This is done by capturing images that are converted to 8-bit grayscale images and then, to black and white using the auto-threshold tool of ImageJ. After conversion, the image is cropped, leaving only the nematodes (black) above a background (white). The total black area in pixels is measured to obtain a nematode count. The problem in using this technique is that the animals’ positions are not always in the same horizontal position. In the same way, an imprecise estimate can occur because this method will also measure the area of eggs, or other developmental stages that are not part of the focus of the estimate. In addition, it would not exclude any dirt or foreign material in the sample. The CEM method does not have the same problems, because the researcher can observe and decide on the developmental stage to be counted; foreign material can similarly be excluded. Therefore, counting errors are less likely with CEM (Fig. 8g and h).

More recently, an automatic counting system for the study of *C. elegans* reproductive aging was proposed by Li et al. [29]. Although not considered an estimator method, because the device actually counts with precision the real number of animals, this method was selected as a comparison. A great advantage of this method is that this automatic microfluidic device is able to detect, count and record in real-time, the progeny production information [29]. Its major disadvantage is its price, which puts it outside the budget of many labs.

Finally, flow cytometry techniques provide precise numbers of animals, and can even provide sorting. Equipment like COPAS™ Biosort can rapidly count a large number of worms in liquid medium with precision [37]. Despite this technique being the “golden standard” of the counting methods, the equipment is expensive and so restrictive for many laboratories [38]. By contrast, CEM uses very cheap materials and equipment that are commonly available in any laboratory. In addition, CEM is adaptable to any environment, including the field.

## Conclusion

This new method was developed because of the low accuracy and poor precision of the conventional method, and as an alternative to expensive automatic high-throughput nematode counting methods. We propose a method that can be accurate, facile, and reproducible and still, less laborious and less expensive than other methods. Perform CEM as an estimation method to nematodes is affordable for practically all laboratories because it requires no expensive devices, uses common materials that every laboratory has and the software for editing images is free of charge. Beyond that, CEM was a better estimator than the conventional DM, with its estimates being closer to those of real counting. Although DM is a faster method, its estimates are not as accurate or as precise as those of CEM.

## Methods

Both methods, CEM and DM, were performed by 3 different operators (Table 1), with different professional background (either master or doctoral level researchers) to assess for operator variation (operator effect). Operator 1 was the first author of this study, while operator 2 and operator 3 were co-authors.

### *Caenorhabditis elegans*

The wild strain N2, Bristol, was cultivated on 3 large agar plates (150 × 21 mm, diameter x height and 147.8 cm^2^ total area) containing nematode growth media (NGM; 3.0 g L^− 1^ NaCl, 5.0 g L^− 1^ peptone, 0.005 g L^− 1^ cholesterol, dissolved in absolute ethanol, 0.11 g L^− 1^ CaCl_2_, 0.12 g L^− 1^ MgSO4, 5.3 g L^− 1^ potassium phosphate, 17.0 g L^− 1^ agar, pH 6.0 at 20 °C, seeded with *Escherichia coli*, strain OP50 as a food source at an optical density at 600 nm = 1.

### Preparation of Nematode Stock Suspension (NSS), Experimental Nematode Suspension (ENS) and Experimental Conditions

A synchronous population of *C. elegans* was obtained after bleaching treatment (50 mL 0.8 M NaOH, 0.5% NaClO) of gravid hermaphrodites cultivated in the 3 culture plates described above. The eggs were incubated until hatching, yielding 10 mL of a very concentrated suspension of nematodes of the same age and similar lengths (homogeneous population). This suspension was stained with 5% Rose Bengal (for enhanced visualization) and incubated for 30 min at 80 °C. This process killed every worm and they assumed a linear morphology. After staining, the suspension was diluted to 30 mL total volume in a 50 mL Falcon tube. This suspension provided the animals used in the different experiments in this study. For each experiment, an aliquot of 5 mL from this suspension was taken and transferred to a 15 mL Falcon tube forming the Nematode Stock Suspension (NSS). Different volumes and densities were created to model environmental and experimental conditions by adding deionized water to the NSS, or by centrifugation and removing a specific volume from the NSS. The total volume sampled from each NSS was called as Experimental Nematode Suspension (ENS).

For each experiment, CEM and DM were performed for comparison by sampling 5 replicates of DM and 1 of CEM (each sample of CEM comprises 4 replicates) Each sample assessed by CEM is a replicate from an ENS, that which in turn, is a replicate from an NSS. To confirm the findings, real counting (RC) of each experiment was also performed.

### Traditional Manual Counting “Drop Method” (DM)

Five replicates of 10 μL of the same ENS were taken at each sampling of CEM and the total nematode content was determined. Prior to taking the aliquots the pipette tip was primed 5 times to avoid clogging. If the nematodes were bigger than 500 μm it was advisable to cut the tip of the pipette as a further precaution against clogging. The pipette tip was then placed right in the middle of the suspension volume to obtain an accurate sampling. The mean count of the 5 replicates was extrapolated to the total volume of the ENS based on the methodology of Solis and Petrascheck [30], adapted according to Scanlan et al.; ensuring shaking of the culture, priming of pipette tips and correct location for sampling within the container [31]. The coefficient of variation (CV) among all replicates from each sampling was recorded to compare the DM and CEM methods. Finally, estimates from both methods were compared with the RC.

### Circular Estimate Method (CEM)

In a dense ENS the animals tended to settle to the bottom of the container. If the area of the container was small, then after a brief homogenization, the animals tended to spread evenly on the bottom (Additional file 1: Figure S1). Photographs could then be taken using a light microscope with a coupled camera (1 photograph if the zoom was capable to catch the entire well or, multiple photographs). Four circles of 1/10 of the total well size were then randomly drawn on the photograph of the well. The total number of worms was counted for each circle and its average extrapolated to the entire well, multiplying by 10. In so doing, extrapolation to the entire ENS and/or NSS volume could be performed.

### Volumes and Samples

The total volume inside the well was 500 μL. This volume was determined after tests of homogenization with 250, 500, 750 and 1000 μL of NSS (Additional file 1: Figure S2). It was found that a better distribution of animals was achieved with 500 μL. Therefore, if the volume that was pipetted into a well was smaller than 500 μL, it was necessary to increase the volume with deionized water.

Different volumes of the NSS were pipetted into a 24 well plate. Each well was treated as a sample; 6 samples were run for each experiment. Several experiments with different ENS volumes pipetted into wells (250, 275, 300, 325, 350, 375, 400, 425, 450, 475 and 500 μL) were performed by all operators (Table 1), and the density of each sample was checked at the end of each experiment (Additional file 1 Table S1). This was done to assess the accuracy, precision, reproducibility, and reliability of both methods, CEM and DM.

### Procedure

A step-by-step protocol of CEM can be accessed in the protocols.io repository (10.17504/protocols.io.qf3dtqn).

After obtained an ENS, the suspension was gently mixed using a vortex, so an aliquot to be estimated could be pipetted into a container. To pipette the aliquot, it was necessary to prime the pipette tip 5 times to avoid clogging. If the nematodes were bigger than 500 μm, it was advisable to cut the tip of the pipette tip as an additional step to avoid clogging. The pipette tip was then placed in the middle of the suspension volume to perform an accurate sampling.

To perform the estimate a well of a 24 cellular culture well plate (TPP, model 92,024) was used. If necessary, the volume was increased to 500 μL. The sample was then homogenized with the same pipette, by pipetting 5 times with the tip located in the centre of the well. It was then necessary to wait until the animals settled to the bottom of the well, approximately 40–60 s.

Photographs were taken, edited and the nematode numbers counted. In our experiments, we used a microscope (Leica, model S8APO) with a coupled camera (Leica, model DFC295). The minimal zoom was not sufficient to cover the total area of a well, so it was necessary to take 4 photographs. These were then joined to cover the entire area. A reference scale was included in the photographs to help with editing. These photographs allowed a count of how many animals were inside a circle corresponding to 1/10 of the total area/volume of the well. For this reason, it was necessary to know the area of the bottom of the well where the ENS would be estimated. According to the manufacturer (TPP), the area of the bottom of a circular well is 1.862 cm^2^; therefore, a circle 1/10 of the total area = 18.62 mm^2^.

With a radius of 2.435 mm, it was possible to draw a circle that could represent a 1/10 of the total area. Such a circle could be drawn with the help of image edition software, ImageJ (Fiji) [39], making use of the tool “Straight” (Fig. 8a) used to measure the length of the scale bar belonging to the photograph. A relationship between the pixel number and a unit of length (1 mm) was made. Using “Set scale” in “Analyze” (Fig. 8b) a numeric value equal to the scale bar was set as mm (Fig. 8c). After calibrating, the “Oval” tool was used to draw the circle of 1/10 diameter (4.87 mm; Fig. 8d, e). By simple computational cutting and pasting the circle could be duplicated to provide 4 circles for counting (circles on the edge) around the central circle (Fig. 8f). The central circle in Fig. 8f was drawn to illustrate the point where the tip has to be located in the homogenization step. Animal counts inside the all 4 circles were made using the “Multi-point” tool (Fig. 8g). Only animals entirely inside the circle were counted (Fig. 8h, i). The counts for the 4 circles were averaged and extrapolated to the entire well or to the entire ENS/NSS.

To confirm the accuracy, precision, reproducibility, and reliability of the method, the rest of animals inside the well were counted, only to confirm the CEM (for the routine estimate, this step was not performed; Additional file 1 Figure S3A). Counting of the number of nematodes in the remaining NSS or ENS to obtain a RC was also performed (Additional file 1 Figure S3B).

The higher RC count for each sample volume was used as a correction factor for the densities discrepancies among the different NSS (marked in red on RC column; Additional file 1 Table S1).

To calculate the module of difference the following formula was used:

1 $$ \boldsymbol{Relative}\ \boldsymbol{difference}\ \boldsymbol{module}=\left|\frac{\boldsymbol{real}\ \boldsymbol{counting}-\boldsymbol{estimate}}{\boldsymbol{real}\ \boldsymbol{counting}}\right| $$

### Biochemical Assay - ROS

An ROS measurement was performed in triplicate in 96 well plates using a hundred animals per sample based on both estimation methods, CEM and DM. To compare the results, 3 controls were included that had a RC of exactly a hundred animals per sample. The total volume of each well plate was adjusted to 90 μL and 10 μL of 10 μM 2′,7′-dichlorodihydrofluorescein diacetate (H_2_DCF-DA; Molecular Probes) was added. The plate was left for 3 h in the dark and after incubation was read over 2 h at 485 and 530 nm for excitation and emission, respectively, to detect ROS. A fluorimeter with microplate reader (Victor 2, PerkinElmer) was used for the ROS measurements, which were expressed as arbitrary units (A.U.).

### Statistics

Statistical data were tested for normality and homoscedasticity by the Shapiro-Wilk and Levene’s tests, respectively [40]. The influence of the methods (CEM or DM) and volumes on CV, count estimates and the module of relative difference were analysed by two-way analysis of variance (ANOVA). The operator effect and ROS measurement were analysed by one-way ANOVA. Both one-way and two-way ANOVA were tested by Newman-Keuls method (*post-hoc*). A significant level of 5% (α = 0.05) was adopted.

## Additional file

Additional file 1: **Figure S1.** Homogenization. (**A**) Image showing nematodes filling the bottom of a 24 culture well plate. (**B**) After a brief homogenization, the nematodes tend to spread evenly over the bottom of the same well. **Figure S2.** Sampled volume determination. Different volumes sampled to achieve the best relationship between photo image resolution and the time required for animals to reach and spread on the bottom of the well. **Figure S3.** The extra step. (**A**) The extra step performed to confirm the ENS real nematode number and (**B**) the number of animals in the exceeding volume. **Table S1:** Comparison of all sample estimates from CEM and DM related to the RC. (**A**) Volumes sampled in range 250–325 μL. (**B**) Volumes sampled in range 350–425 μL. (**C**) Volumes sampled in range 450–500 μL. First column: the sample volume assessed by each operator. Second column: the operator (OP 1, OP 2 or OP 3) responsible for each experiment. Third column: the order of samples. Fourth column: number of nematodes estimated by CEM. Fifth column: number of nematodes counted by RC; the number marked in red is the highest count and was used as the correction index. Sixth column: number of nematodes estimated by DM. Seventh column: the corrected CEM. Eighth column: the corrected RC. Ninth column: the corrected DM. (DOCX 3180 kb)

## Abbreviations

CEM: Circular estimate method
CV: Coefficient of variation
DM: Drop method
ENS: Experimental nematode suspension
NSS: Nematode stock suspension
OP: Operator
RC: Real counting
ROS: Reactive oxygen species
SI: Supporting information
