# Supplementary material for: Circular Estimate Method (CEM) - a Simple Method to Estimate Caenorhabditis elegans Culture Densities in Liquid Medium
Source: Biol Proced Online. 2019 Jan 15;21:1. doi: 10.1186/s12575-018-0089-2 (PMC6334471; doi:10.1186/s12575-018-0089-2)
Supplement: Supplementary file 1 — Figure S1. Homogenization. (A) Image showing nematodes filling the bottom of a 24 culture well plate. (B) After a brief homogenization, the nematodes tend to spread evenly over the bottom of the same well. Figure S2. Sampled volume determination. Different volumes sampled to achieve the best relationship between photo image resolution and the time required for animals to reach and spread on the bottom of the well. Figure S3. The extra step. (A) The extra step performed to confirm the ENS real nematode number and (B) the number of animals in the exceeding volume. Table S1: Comparison of all sample estimates from CEM and DM related to the RC. (A) Volumes sampled in range 250–325 μL. (B) Volumes sampled in range 350–425 μL. (C) Volumes sampled in range 450–500 μL. First column: the sample volume assessed by each operator. Second column: the operator (OP 1, OP 2 or OP 3) responsible for each experiment. Third column: the order of samples. Fourth column: number of nematodes estimated by CEM. Fifth column: number of nematodes counted by RC; the number marked in red is the highest count and was used as the correction index. Sixth column: number of nematodes estimated by DM. Seventh column: the corrected CEM. Eighth column: the corrected RC. Ninth column: the corrected DM. (DOCX 3180 kb) [file 12575_2018_89_MOESM1_ESM.docx]

Supporting Information

**Circular Estimate Method (CEM) - A Simple Method to Estimate** *Caenorhabditis elegans* **Culture Densities in Liquid Medium.**

Marcelo Estrella Josende^1,2,*^, Silvana Manske Nunes ^1,2^, Larissa Müller ^1,2^, Marlize Ferreira-Cravo^1^, José Marìa Monserrat^1,2^ and Juliane Ventura-Lima^1,2^

^1^Instituto de Ciências Biológicas (ICB), Universidade Federal do Rio Grande - FURG, Av. Itália km 8, CEP: 96203-900. Rio Grande, RS, Brazil.

^2^Programa de Pós-Graduação em Ciências Fisiológicas (PPGCF) - FURG.

* Corresponding author.

E-mail: address: [marcelo.e.josende@gmail.com](mailto:marcelo.e.josende@gmail.com) (MEJ)


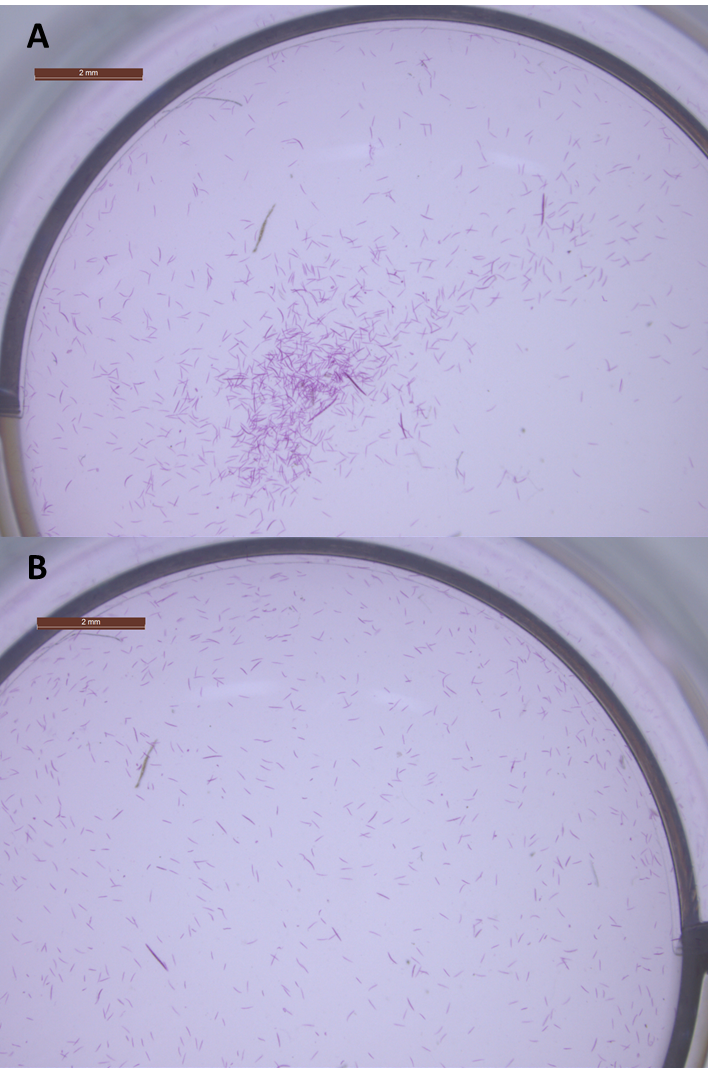


**Fig. S1** Homogenization. (**A**) Image showing nematodes filling the bottom of a 24 culture well plate. (**B**) After a brief homogenization, the nematodes tend to spread evenly over the bottom of the same well.


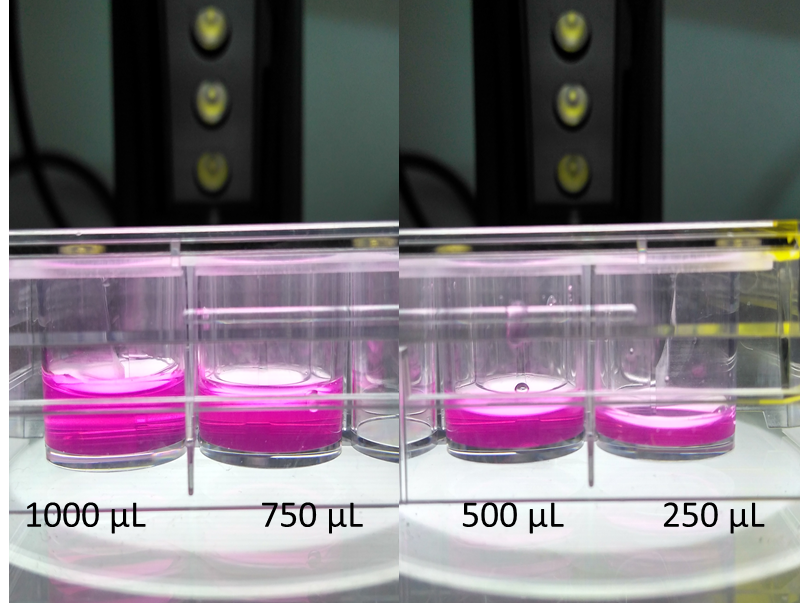


**Fig. S2** Sampled volume determination. Different volumes sampled to achieve the best relationship between photo image resolution and the time required for animals to reach and spread on the bottom of the well.


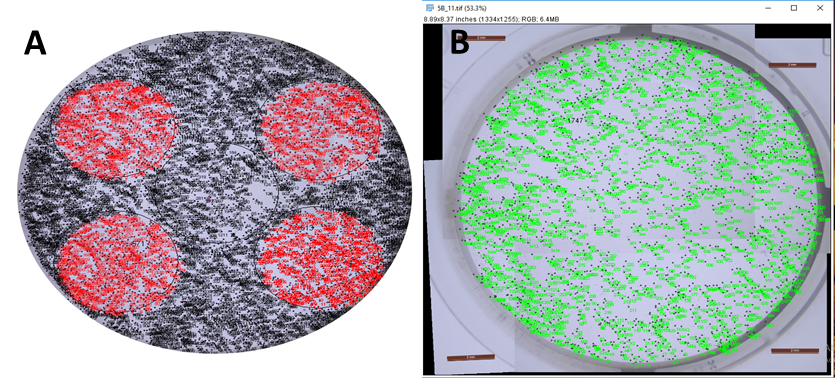


**Fig. S3** The extra step. (**A**) The extra step performed to confirm the ENS real nematode number and (**B**) the number of animals in the exceeding volume.

Table S1_A

| **Sampled volume** | **Operator** | **Sample** | **CEM** | **RC** | **DM** | **CEM*** | **RC*** | **DM*** |
| --- | --- | --- | --- | --- | --- | --- | --- | --- |
|  |  | 1 | 2917,5 | **3058** | 3205,0 | 2917,5 | 3058,0 | 3205,0 |
|  |  | 2 | 2930,0 | **2873** | 3650,0 | 3118,7 | 3058,0 | 3885,0 |
|  | OP 1 | 3 | 2970,0 | **3034** | 3390,0 | 2993,5 | 3058,0 | 3416,8 |
|  |  | 4 | 2967,5 | **3011** | 3210,0 | 3013,8 | 3058,0 | 3260,1 |
|  |  | 5 | 3057,5 | **2965** | 3300,0 | 3153,4 | 3058,0 | 3403,5 |
| 250 µL |  | 6 | 2952,5 | **2887** | 3110,0 | 3127,4 | 3058,0 | 3294,2 |
|  |  | 1 | 2750,0 | **2699** | 3185,0 | 3115,8 | 3058,0 | 3608,6 |
|  |  | 2 | 2907,5 | **2781** | 3970,0 | 3197,1 | 3058,0 | 4365,4 |
|  | OP 3 | 3 | 2735,0 | **2839** | 3380,0 | 2946,0 | 3058,0 | 3640,7 |
|  |  | 4 | 2652,5 | **2714** | 2910,0 | 2988,7 | 3058,0 | 3278,8 |
|  |  | 5 | 2535,0 | **2496** | 3390,0 | 3105,8 | 3058,0 | 4153,3 |
|  |  | 6 | 2447,5 | **2483** | 3290,0 | 3014,3 | 3058,0 | 4051,9 |
|  |  | 1 | 3147,5 | **3276** | 3459,5 | 3257,0 | 3390,0 | 3579,9 |
|  |  | 2 | 3350,0 | **3307** | 3492,5 | 3434,1 | 3390,0 | 3580,2 |
|  | OP 1 | 3 | 3175,0 | **3284** | 3597,0 | 3277,5 | 3390,0 | 3713,1 |
|  |  | 4 | 3395,0 | **3296** | 3492,5 | 3491,8 | 3390,0 | 3592,1 |
|  |  | 5 | 3342,5 | **3223** | 3459,5 | 3515,7 | 3390,0 | 3638,8 |
| 275 µL |  | 6 | 3347,5 | **3390** | 3608,0 | 3347,5 | 3390,0 | 3608,0 |
|  |  | 1 | 1312,5 | **1225** | 1958,0 | 3632,1 | 3390,0 | 5418,5 |
|  |  | 2 | 1320,0 | **1351** | 1969,0 | 3312,2 | 3390,0 | 4940,7 |
|  | OP 3 | 3 | 1357,5 | **1267** | 2183,5 | 3632,1 | 3390,0 | 5842,2 |
|  |  | 4 | 1380,0 | **1201** | 2376,0 | 3895,3 | 3390,0 | 6706,6 |
|  |  | 5 | 1387,5 | **1246** | 2315,5 | 3775,0 | 3390,0 | 6299,8 |
|  |  | 6 | 1280,0 | **1259** | 2073,5 | 3446,5 | 3390,0 | 5583,1 |
|  |  | 1 | 3487,5 | **3524** | 3600,0 | 3648,8 | 3687,0 | 3766,5 |
|  |  | 2 | 3520,0 | **3632** | 3804,0 | 3573,3 | 3687,0 | 3861,6 |
|  | OP 1 | 3 | 3502,5 | **3461** | 3798,0 | 3731,2 | 3687,0 | 4046,0 |
|  |  | 4 | 3525,0 | **3687** | 4020,0 | 3525,0 | 3687,0 | 4020,0 |
|  |  | 5 | 3335,0 | **3402** | 3990,0 | 3614,4 | 3687,0 | 4324,3 |
| 300 µL |  | 6 | 3455,0 | **3596** | 3858,0 | 3542,4 | 3687,0 | 3955,6 |
|  |  | 1 | 2265,0 | **2259** | 2472,0 | 3696,8 | 3687,0 | 4034,6 |
|  |  | 2 | 2017,5 | **1962** | 2232,0 | 3791,3 | 3687,0 | 4194,4 |
|  | OP 2 | 3 | 2070,0 | **2311** | 2514,0 | 3302,5 | 3687,0 | 4010,9 |
|  |  | 4 | 2175,0 | **2210** | 2406,0 | 3628,6 | 3687,0 | 4014,0 |
|  |  | 5 | 2205,0 | **2141** | 2430,0 | 3797,2 | 3687,0 | 4184,7 |
|  |  | 6 | 2257,5 | **2179** | 2670,0 | 3819,8 | 3687,0 | 4517,8 |
|  |  | 1 | 3782,5 | **3878** | 4342,0 | 4097,5 | 4201,0 | 4703,6 |
|  |  | 2 | 3820,0 | **3819** | 4439,5 | 4202,1 | 4201,0 | 4883,6 |
|  | OP 1 | 3 | 3837,5 | **3749** | 4459,0 | 4300,2 | 4201,0 | 4996,6 |
|  |  | 4 | 3827,5 | **3961** | 4407,0 | 4059,4 | 4201,0 | 4674,0 |
|  |  | 5 | 3770,0 | **3914** | 4309,5 | 4046,4 | 4201,0 | 4625,5 |
| 325 µL |  | 6 | 3772,5 | **3899** | 4673,5 | 4064,7 | 4201,0 | 5035,5 |
|  |  | 1 | 4012,5 | **3892** | 4173,0 | 4331,1 | 4201,0 | 4504,3 |
|  |  | 2 | 4020,0 | **3866** | 4062,5 | 4368,3 | 4201,0 | 4414,5 |
|  | OP 2 | 3 | 4130,0 | **4158** | 4283,5 | 4172,7 | 4201,0 | 4327,8 |
|  |  | 4 | 4055,0 | **4127** | 4264,0 | 4127,7 | 4201,0 | 4340,5 |
|  |  | 5 | 4112,5 | **4201** | 3991,0 | 4112,5 | 4201,0 | 3991,0 |
|  |  | 6 | 3986,7 | **3833** | 4147,0 | 4369,4 | 4201,0 | 4545,1 |

Table S1_B

| **Sampled volume** | **Operator** | **Sample** | **CEM** | **RC** | **DM** | **CEM*** | **RC*** | **DM*** |
| --- | --- | --- | --- | --- | --- | --- | --- | --- |
|  |  | 1 | 2715,0 | **2764** | 2898,0 | 3731,7 | 3799,0 | 3983,2 |
|  |  | 2 | 2735,0 | **2677** | 3003,0 | 3881,3 | 3799,0 | 4261,6 |
|  | OP 1 | 3 | 2742,5 | **2802** | 3164,0 | 3718,3 | 3799,0 | 4289,8 |
|  |  | 4 | 2650,0 | **2741** | 2933,0 | 3672,9 | 3799,0 | 4065,1 |
|  |  | 5 | 2725,0 | **2626** | 3087,0 | 3942,2 | 3799,0 | 4465,9 |
| 350 µL |  | 6 | 2737,5 | **2797** | 3059,0 | 3718,2 | 3799,0 | 4154,9 |
|  |  | 1 | 3947,5 | **3799** | 5166,0 | 3947,5 | 3799,0 | 5166,0 |
|  |  | 2 | 3580,0 | **3650** | 5397,0 | 3726,1 | 3799,0 | 5617,3 |
|  | OP 3 | 3 | 3450,0 | **3714** | 5166,0 | 3529,0 | 3799,0 | 5284,2 |
|  |  | 4 | 3615,0 | **3761** | 5320,0 | 3651,5 | 3799,0 | 5373,8 |
|  |  | 5 | 3615,0 | **3594** | 5180,0 | 3821,2 | 3799,0 | 5475,5 |
|  |  | 6 | 3677,5 | **3476** | 5306,0 | 4019,2 | 3799,0 | 5799,0 |
|  |  | 1 | 2822,5 | **2903** | 3262,5 | 2918,8 | 3002,0 | 3373,8 |
|  |  | 2 | 2957,5 | **2887** | 3592,5 | 3075,3 | 3002,0 | 3735,6 |
|  | OP 1 | 3 | 2902,5 | **3002** | 3390,0 | 2902,5 | 3002,0 | 3390,0 |
|  |  | 4 | 2745,0 | **2947** | 3352,5 | 2796,2 | 3002,0 | 3415,1 |
|  |  | 5 | 2757,5 | **2890** | 3465,0 | 2864,4 | 3002,0 | 3599,3 |
| 375 µL |  | 6 | 2807,5 | **2983** | 3577,5 | 2825,4 | 3002,0 | 3600,3 |
|  |  | 1 | 2522,5 | **2646** | 3330,0 | 2861,9 | 3002,0 | 3778,0 |
|  |  | 2 | 2470,0 | **2659** | 3262,5 | 2788,6 | 3002,0 | 3683,3 |
|  | OP 2 | 3 | 2647,5 | **2716** | 3022,5 | 2926,3 | 3002,0 | 3340,8 |
|  |  | 4 | 2542,5 | **2493** | 3172,5 | 3061,6 | 3002,0 | 3820,2 |
|  |  | 5 | 2650,0 | **2597** | 3180,0 | 3063,3 | 3002,0 | 3675,9 |
|  |  | 6 | 2520,0 | **2445** | 3202,5 | 3094,1 | 3002,0 | 3932,1 |
|  |  | 1 | 2840,0 | **2707** | 3840,0 | 5258,2 | 5012,0 | 7109,7 |
|  |  | 2 | 3187,5 | **3091** | 3752,0 | 5168,5 | 5012,0 | 6083,8 |
|  | OP 1 | 3 | 3222,5 | **3036** | 3928,0 | 5319,9 | 5012,0 | 6484,6 |
|  |  | 4 | 3105,0 | **2955** | 3632,0 | 5266,4 | 5012,0 | 6160,3 |
|  |  | 5 | 2977,5 | **3036** | 3456,0 | 4915,4 | 5012,0 | 5705,4 |
| 400 µL |  | 6 | 2830,0 | **2955** | 3224,0 | 4800,0 | 5012,0 | 5468,3 |
|  |  | 1 | 4617,5 | **4625** | 5880,0 | 5003,9 | 5012,0 | 6372,0 |
|  |  | 2 | 4535,0 | **4651** | 6064,0 | 4887,0 | 5012,0 | 6534,7 |
|  | OP 2 | 3 | 4767,5 | **5012** | 5960,0 | 4767,5 | 5012,0 | 5960,0 |
|  |  | 4 | 4692,5 | **4834** | 6400,0 | 4865,3 | 5012,0 | 6635,7 |
|  |  | 5 | 4850,0 | **4926** | 5912,0 | 4934,7 | 5012,0 | 6015,2 |
|  |  | 6 | 4665,0 | **4952** | 6424,0 | 4721,5 | 5012,0 | 6501,8 |
|  |  | 1 | 5820,0 | **5820** | 8636,0 | 6115,0 | 6115,0 | 9073,7 |
|  |  | 2 | 6132,5 | **5758** | 7803,0 | 6512,7 | 6115,0 | 8286,8 |
|  | OP 1 | 3 | 5437,5 | **5470** | 8117,5 | 6078,7 | 6115,0 | 9074,7 |
|  |  | 4 | 5750,0 | **5903** | 8160,0 | 5956,5 | 6115,0 | 8453,1 |
|  |  | 5 | 5792,5 | **5847** | 7726,5 | 6058,0 | 6115,0 | 8080,6 |
| 425 µL |  | 6 | 5900,0 | **6115** | 7905,0 | 5900,0 | 6115,0 | 7905,0 |
|  |  | 1 | 5205,0 | **4958** | 5584,5 | 6419,6 | 6115,0 | 6887,7 |
|  |  | 2 | 5347,5 | **5143** | 5431,5 | 6358,1 | 6115,0 | 6458,0 |
|  | OP 2 | 3 | 5232,5 | **5101** | 5686,5 | 6272,6 | 6115,0 | 6816,9 |
|  |  | 4 | 5225,0 | **5006** | 5584,5 | 6382,5 | 6115,0 | 6821,7 |
|  |  | 5 | 5302,5 | **5234** | 5601,5 | 6195,0 | 6115,0 | 6544,4 |
|  |  | 6 | 5287,5 | **5316** | 5550,5 | 6082,2 | 6115,0 | 6384,7 |

Table S1_C

| **Sampled volume** | **Operator** | **Sample** | **CEM** | **RC** | **DM** | **CEM*** | **RC*** | **DM*** |
| --- | --- | --- | --- | --- | --- | --- | --- | --- |
|  |  | 1 | 6257,5 | **6348** | 8145,0 | 6828,2 | 6927,0 | 8887,9 |
|  |  | 2 | 6475,0 | **6386** | 8388,0 | 7023,5 | 6927,0 | 9098,6 |
|  | OP 1 | 3 | 6677,5 | **6927** | 8361,0 | 6677,5 | 6927,0 | 8361,0 |
|  |  | 4 | 6472,5 | **6646** | 8253,0 | 6746,2 | 6927,0 | 8601,9 |
|  |  | 5 | 6420,0 | **6592** | 8748,0 | 6746,3 | 6927,0 | 9192,6 |
| 450 µL |  | 6 | 6575,0 | **6723** | 8208,0 | 6774,5 | 6927,0 | 8457,1 |
|  |  | 1 | 4625,0 | **4555** | 5760,0 | 7033,5 | 6927,0 | 8759,5 |
|  |  | 2 | 4472,5 | **4267** | 5625,0 | 7260,6 | 6927,0 | 9131,6 |
|  | OP 3 | 3 | 4642,5 | **4398** | 5517,0 | 7312,1 | 6927,0 | 8689,5 |
|  |  | 4 | 4720,0 | **4662** | 5940,0 | 7013,2 | 6927,0 | 8825,9 |
|  |  | 5 | 4477,5 | **4801** | 5184,0 | 6460,2 | 6927,0 | 7479,6 |
|  |  | 6 | 4667,5 | **4731** | 6075,0 | 6834,0 | 6927,0 | 8894,8 |
|  |  | 1 | 6372,5 | **6141** | 8380,0 | 7184,0 | 6923,0 | 9447,1 |
|  |  | 2 | 5720,0 | **5566** | 9910,0 | 7114,5 | 6923,0 | 12326,1 |
|  | OP 1 | 3 | 6727,5 | **6654** | 9770,0 | 6999,5 | 6923,0 | 10165,0 |
|  |  | 4 | 6632,5 | **6923** | 9970,0 | 6632,5 | 6923,0 | 9970,0 |
|  |  | 5 | 6585,0 | **6751** | 9690,0 | 6752,8 | 6923,0 | 9936,9 |
| 475 µL |  | 6 | 6757,5 | **6892** | 9870,0 | 6787,9 | 6923,0 | 9914,4 |
|  |  | 1 | 3095,0 | **3037** | 3553,0 | 7055,2 | 6923,0 | 8099,2 |
|  |  | 2 | 3092,5 | **3656** | 4322,5 | 5856,0 | 6923,0 | 8185,1 |
|  | OP 3 | 3 | 3120,0 | **3171** | 3705,0 | 6811,7 | 6923,0 | 8088,8 |
|  |  | 4 | 2797,5 | **2819** | 3686,0 | 6870,2 | 6923,0 | 9052,2 |
|  |  | 5 | 2780,0 | **2804** | 3420,0 | 6863,7 | 6923,0 | 8443,9 |
|  |  | 6 | 2752,5 | **2967** | 3876,0 | 6422,5 | 6923,0 | 9044,0 |
|  |  | 1 | 3007,5 | **2949** | 4290,0 | 6327,1 | 6204,0 | 9025,1 |
|  |  | 2 | 3022,5 | **2863** | 4380,0 | 6549,6 | 6204,0 | 9491,3 |
|  | OP 1 | 3 | 2825,0 | **2778** | 4070,0 | 6309,0 | 6204,0 | 9089,4 |
|  |  | 4 | 3157,5 | **3022** | 4490,0 | 6482,2 | 6204,0 | 9217,7 |
|  |  | 5 | 3075,0 | **2913** | 5740,0 | 6549,0 | 6204,0 | 12224,8 |
| 500 µ |  | 6 | 3342,5 | **2988** | 4170,0 | 6940,1 | 6204,0 | 8658,2 |
|  |  | 1 | 5762,5 | **5944** | 7640,0 | 6014,6 | 6204,0 | 7974,2 |
|  |  | 2 | 5910,0 | **5801** | 7650,0 | 6320,6 | 6204,0 | 8181,5 |
|  | OP 2 | 3 | 5592,5 | **5723** | 7080,0 | 6062,5 | 6204,0 | 7675,1 |
|  |  | 4 | 5835,0 | **5993** | 7540,0 | 6040,4 | 6204,0 | 7805,5 |
|  |  | 5 | 5965,0 | **6204** | 7080,0 | 5965,0 | 6204,0 | 7080,0 |
|  |  | 6 | 5737,5 | **5849** | 7460,0 | 6085,7 | 6204,0 | 7912,8 |

**Table S1** Comparison of all sample estimates from CEM and DM related to the RC. (**A**) Volumes sampled in range 250 – 325 µL. (**B**) Volumes sampled in range 350 – 425 µL. (**C**) Volumes sampled in range 450 – 500 µL. First column: the sample volume assessed by each operator. Second column: the operator (OP 1, OP 2 or OP 3) responsible for each experiment. Third column: the order of samples. Fourth column: number of nematodes estimated by CEM. Fifth column: number of nematodes counted by RC; the number marked in red is the highest count and was used as the correction index. Sixth column: number of nematodes estimated by DM. Seventh column: the corrected CEM. Eighth column: the corrected RC. Ninth column: the corrected DM.
